# Liposomal Iron Oxide Nanoparticles Loaded with Doxorubicin for Combined Chemo-Photothermal Cancer Therapy

**DOI:** 10.3390/pharmaceutics15010292

**Published:** 2023-01-15

**Authors:** Taehoon Park, Reeju Amatya, Kyoung Ah Min, Meong Cheol Shin

**Affiliations:** 1 College of Pharmacy and Research Institute of Pharmaceutical Sciences, Gyeongsang National University, 501 Jinju Daero, Jinju 52828, Gyeongnam, Republic of Korea; 2 College of Pharmacy and Inje Institute of Pharmaceutical Sciences and Research, Inje University, 197 Injero, Gimhae 50834, Gyeongnam, Republic of Korea

**Keywords:** iron oxide nanoparticle, liposome, magnetic targeting, photothermal therapy, cancer, doxorubicin

## Abstract

Iron oxide nanoparticle (IONP) possesses unique advantages over other nanoparticles in the use of cancer imaging and therapy. Specifically, it has drawn great attention in the emerging research field of photothermal cancer therapy. Herein, we developed doxorubicin (DOX)-loaded liposomal IONP (Lipo-IONP/DOX) and evaluated in vitro and in vivo their applicability for combined chemo-photothermal cancer therapy. The Lipo-IONP was synthesized by the thin-film evaporation method. The prepared Lipo-IONP was observed as about a 240 nm-sized agglomerate of globular-shaped nanoparticles. The TEM and FT-IR data evidenced the successful formation of liposomal IONP. The superparamagnetic property of the Lipo-IONP was confirmed by the SQUID analysis. The DSC data showed a transition temperature of about 47–48 °C for the mixed lipids composing the Lipo IONP, and the DOX release studies revealed the feasibility of induced burst release of DOX by laser irradiation. The Lipo-IONP/DOX possessed a plasma half-life of 42 min, which could ensure sufficient circulation time for magnetic tumor targeting. The in vivo magnetic targeting enabled a significant increase (6.3-fold) in the tumor accumulation of Lipo-IONP/DOX, leading to greater photothermal effects. Finally, the preliminary efficacy study evidenced the applicability as well as the safety of the Lipo-IONP/DOX for use in combined chemo-photothermal cancer therapy. Overall, the study results demonstrated that the Lipo-IONP/DOX might serve as an effective and safe agent for combined chemo-photothermal cancer therapy.

## 1. Introduction

Photothermal therapy (PTT) has gained great attention as a promising modality for cancer treatment [1,2]. It is carried out by focal laser irradiation to the tumor region after a photosensitive agent is delivered to the target site [1,3]. By conversion of the absorbed light to heat, hyperthermic cell death is induced at temperatures above mid-40 °C in the tumor via various cell death mechanisms that include necrosis, necroptosis, or apoptosis [4]. Apart from directly killing the tumor or tumor vasculature cells, the PTT could also be used for combination therapies by enhancing the tumor blood flow, triggering the release, or enhancing the tumor cell uptake of chemotherapeutics [5]. Specifically, with the discovery of photoactive nanomaterials (such as IONP), these combination therapies are gaining more interest for obtaining synergistic or additive therapeutic effects in the cancer research field [3].

Despite extensive research efforts to reach clinical application, there yet remain several bottleneck challenges: one is the limited penetration depth of light inside the human body, and the other is the effective heat confinement in the tumor [5]. Regarding the penetration issue, the use of light in the NIR wavelength is favored for its greater penetration depth which could reach a few centimeters under the skin [6]. To gain further access to deeper region tumors, the intervention of a surgical method may also be beneficial [7]. To resolve the heat confinement challenge, effective tumor targeting of the PTT agent is necessary, and, in this regard, the use of IONP as the PTT agent may become a great option.

The IONP is a core-shell type nanoparticle [8]. It comprises an iron oxide core and a hydrophilic coating [8]. Compared with other metal-based nanoparticles available for PTT agents, the IONP possesses several unique advantages. First, it is a clinically approved MRI agent (Feridex^®^) for liver imaging and also a therapeutics (Feraheme^®^) for the treatment of iron deficiency [9]. Unlike other particles, the safety of the clinical use of IONP has been ensured. Second, the IONP is superparamagnetic, which means it could be externally directed to a certain site using a magnet [10]. In recent years, there have been accumulating reports that evidenced the feasibility of magnetic tumor targeting of the IONP in animal models [11]. For example, Zhang et al. reported that, in an *s.c.* tumor mouse model, magnetic targeting could provide a markedly higher (5-fold) tumor accumulation of the IONP compared with passive targeting [12]. Lastly, the IONP possesses a large heat conversion capacity when applying an alternating magnetic field or a laser. Thus, IONP has long been used in various hyperthermia research [13].

Because of the poor water solubility of the iron oxide core, proper coating of the particles is a critical issue [14]. To ensure safety and stability, the coating material should be biodegradable and biocompatible [14]. Furthermore, it would be beneficial for combination therapy if the coating process allows efficient drug encapsulation. In this regard, developing a liposomal formulation of IONP could be an effective strategy to resolve these issues. The liposome is a lipid-based nanoparticle that has been clinically used for the safer delivery of chemotherapeutics [15]. The lipids are biocompatible and biodegradable [16]. Composed of a water (or buffer)-filled core and an amphiphilic double-layered membrane, both hydrophilic and hydrophobic drugs could be loaded into the liposome [15]. Due to these merits, the liposome could serve as a carrier for the co-delivery of hydrophilic and hydrophobic drugs. Further, there have also been reports of successful encapsulation of small nanoparticles into the liposomes [17,18].

**Figure 1:**
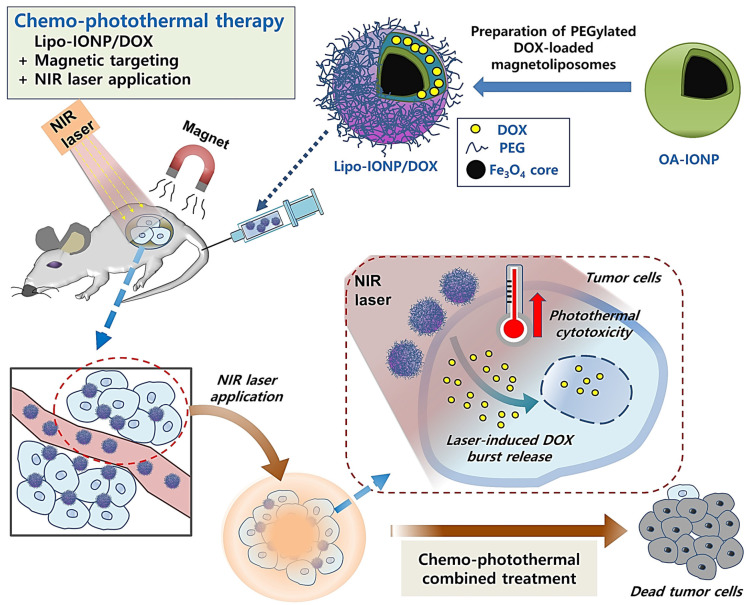
Scheme of the doxorubicin-loaded liposomal iron oxide nanoparticles for magnet-guided and light-triggered combined chemo-photothermal cancer therapy.

In this research, we developed Lipo-IONP and characterized its physicochemical and photothermal properties. Then, IONP and a chemotherapeutics, doxorubicin (DOX), were co-loaded to liposomes, and, after preparation of the Lipo-IONP/DOX, the stability, drug loading, and release profiles were determined. The applicability of Lipo-IONP/DOX in combined chemo-photothermal therapy was evaluated both in vitro and in vivo (Figure 1).

## 2. Materials and Methods

### 2.1. Materials

Oleic acid-coated iron oxide nanoparticle (OA-IONP) was purchased from Ocean NanoTech (San Diego, CA, USA). 1,2-dipalmitoyl-sn-glycero-3-phosphocholine (DPPC) was purchased from Avanti Polar Lipids (Alabaster, AL, USA). mPEG-DSPE (DSPE-P2000; MW: 2000 Da) was purchased from Creative PEGWorks (Chapel Hill, NC, USA). DOX was purchased from Sigma-Aldrich (St. Louis, MO, USA).

### 2.2. Synthesis of Lipo-IONP

The Lipo-IONP was prepared using the standard thin-film hydration method [19,20]. Briefly, a fixed amount of DPPC was mixed together with DSPE-P2000 at different molar ratios of 3:1, 4:1, or 5:1 (for L1, L2, and L3, respectively) in a total of 1 mL chloroform. These mix ratios were decided based on the lipid mixtures’ theoretical transition temperatures to implement burst release of the encapsulated drugs when the tumor region was heated up to about 45–50 °C by laser irradiation. Then OA-IONP 1 mg Fe (dispersed in chloroform) was added. For example, for L3, 2.5 mg of DPPC and 1.9 mg of DSPE-P2000 were dissolved in chloroform, and then 1 mg of OA-IONP (40 μL of 25 mgFe/mL dispersed in chloroform) was added. After incubation for 10 min at room temperature (RT) with shaking, the solvent was evaporated under reduced pressure, and the Lipo-IONP lipid film was hydrated in 1 mL of pre-warmed (60 °C) double distilled water (DDW). The prepared Lipo-IONP was filtered through a syringe filter (pore size: 0.4 μm diameter) to remove the nonencapsulated OA-IONP and then extruded against a 0.4 μm diameter pore size membrane. The final product of Lipo-IONP was stored at 4 °C before use. For DOX loading, the lipid film was hydrated with 1 mL of a pre-warmed (60 °C) DOX-containing solution and then, after filtration with a syringe filter (pore size: 0.4 μm diameter), centrifuged at 30,000× *g* for 30 min. The supernatant containing the unloaded DOX was removed, and the particles were dispersed in PBS. The Lipo-IONP/DOX was then extruded against a 0.4 μm diameter pore size membrane and stored at 4 °C before use.

### 2.3. Physical Characterization of Lipo-IONP

The iron content of the Lipo-IONP was quantified by inductively coupled plasma optical emission spectroscopy (ICP−OES) (Perkin Elmer, Norwalk, CT, USA), and the presence of lipid coating and the PEG on the surface of the particles was identified by FT-IR spectra using a spectrometer (VERTEX 80v, wavelength range: 400–4000 cm^−1^; Bruker, Billerica, MA, USA). The PEG contents associated with the particles were quantified using the barium iodide assay. The hydrodynamic diameters and zeta potentials of Lipo-IONP were analyzed by DLS (Zetasizer Nano ZS, Malvern Panalytical Ltd., Malvern, UK), and the morphology was examined using transmission electron microscopy (TEM; TF30ST, FEI, Hillsboro, OR, USA) operating at 300 kV. The size stability of Lipo-IONP was monitored by DLS for 5 days. The magnetization measurement for OA-IONP and Lipo-IONP was carried out using the MPMS-XL SQUID magnetometer (Quantum Design Inc. San Diego, CA, USA). Briefly, pulverized particle samples were suspended in an eicosane matrix, loaded in capsules, and then analyzed under a magnetic field (0–30,000 Oe). The differential scanning calorimetry (DSC) analysis was performed for each type of Lipo-IONP (L1, L2, or L3) with the different molar ratios (3:1, 4:1, or 5:1 of DPPC:DSPE-P2000, respectively). TA Instruments Q20 DSC (New Castle, DE, USA) was used for the measurement with the heating rate of 10 °C/min under nitrogen gas purging conditions.

### 2.4. Measurement of the Photothermal Activity of Lipo-IONP

To verify the photothermal conversion capacity, the Lipo-IONP suspensions were prepared in Eppendorf tubes (at a fixed concentration of 150 μgFe/mL) and then irradiated with a diode laser (885 nm, spot size, 5 × 8 mm^2^, MDL-III-885, Changchun New Industries Optoelectronics Tech Co. Ltd., Changchun, China) at varying laser powers (0.7–1.5 W) for 10 min per each. On the other hand, the Lipo-IONP suspensions were also prepared at varying concentrations (0, 25, 50, 100, 150, or 200 μgFe/mL) in Eppendorf tubes and irradiated with the laser (885 nm) at a fixed laser power of 1.3 W for 10 min per each. In addition, to assess the photostability of the Lipo-IONP, the Lipo-IONP suspension (150 μgFe/mL) was irradiated by the laser at a switch on and off mode (a total of 3 cycles of laser switch “on” for 10 min followed by “off” for 10 min) at a laser power of 1.3 W. The temperature profiles of the Lipo-IONP suspensions were monitored using an infrared (IR) camera (FLIR Systems, E5, Boston, MA, USA).

### 2.5. DOX Loading and Release from Lipo-IONP

Lipo-IONP/DOX samples were prepared with the addition of different amounts of DOX (0, 50, 100, 200, or 400 μg) to the Lipo-IONP during the hydration process of Lipo-IONP synthesis. The prepared Lipo-IONP/DOX was centrifuged (30,000× *g* × 30 min), and the unloaded DOX content in the supernatant was measured by UV/VIS spectrophotometry (at 490 nm wavelength). The particle-loaded DOX content was calculated by subtracting the amounts of unloaded DOX from the initial fed DOX.

To assess the DOX release profiles, the Lipo-IONP/DOX dispersed in PBS (pH 7.4) was added to 20 mL glass vials (5 mL per vial) and divided into 2 groups. For one group, the vial was irradiated with a laser for 10 min at 0.7 W (maximum suspension temperature: 48 °C), and, for the other group, kept at RT without laser irradiation. Afterward, the Lipo-IONP/DOX samples were placed in a dialysis bag (MWCO 14 kDa; dialysis tubing cellulose membrane; Sigma Aldrich, St. Louis. MO, USA) and dialyzed against 50 mL of PBS at 37 °C by gentle agitation. At pre-determined time points (0, 1, 2, 4, 6, 10, 24, and 48 h), 1 mL of the suspension was collected, and the same volume of fresh PBS buffer was added. The released DOX contents were quantified by UV/VIS spectrophotometry (at 490 nm).

### 2.6. Cell Culture

The B16F10 murine melanoma cell line was purchased from the Korean cell line bank (KCLB, Seoul, Republic of Korea). The B16F10 cells were cultured in a DMEM medium (with 10% fetal bovine serum, 1% antibiotic antimycotic, and 1% penicillin-streptomycin), and the cell culture was maintained in a humidified CO_2_ cell incubator.

### 2.7. Cytotoxicity of Lipo-IONP/DOX

To evaluate the cytotoxicity of Lipo-IONP/DOX, the B16F10 cells were seeded onto 96-well plates (5 × 10^3^ cells/well). After incubation overnight, the cells were treated with either 1) PBS, DOX, Lipo-IONP, or Lipo-IONP/DOX. The DOX and Lipo-IONP/DOX samples were added at varying concentrations (10^−10^–10^−4^ M as DOX). After treatment, the cells were further incubated for 48 h, and then the relative cell viability was determined by the WST-1 assay ((iNtRON Biotechnology, Daejeon, Republic of Korea). In addition, to assess the photothermal cytotoxicity of Lipo-IONP/DOX, the B16F10 cells plated on 96-well plates (5 × 10^3^ cells/well) were separately treated with PBS, DOX (5 μM), Lipo-IONP (150 μgFe/mL), and Lipo-IONP/DOX (150 μgFe/mL and 5 μM as DOX). For a group of sample-treated cells, the laser was irradiated for 10 min with differential laser outputs (0.9, 1.1, and 1.3 W) to acquire maximum medium temperatures of about 40, 45, and 50 °C, respectively.

### 2.8. Animal Studies

Every animal experiment was conducted by the National Institute of Health Guidelines on the Use of Laboratory Animals, and the protocol was approved by the university’s committee for animal research (GNU-180724-M0037).

#### 2.8.1. Pharmacokinetics (PK)

Ten healthy ICR mice (20 ± 1.4 g) were administered with either PBS or Lipo-IONP/DOX (6 mgFe/kg; 1 mg/kg DOX) via the tail vein. After injection, blood was collected at pre-determined time points (0, 10 min, 0.5, 1, 1.5, 2, and 4 h post-administration), and plasma samples were acquired by centrifugation of the blood. The iron contents in the plasma samples were quantified by ICP-OES analyses. The acquired iron contents were further subtracted by those from the control mice plasma and then plotted against the time.

#### 2.8.2. Tissue Distribution

Tissue distribution of the Lipo-IONP/DOX was assessed in B16F10 *s.c.* tumor-bearing mice. The mice model was prepared by subcutaneous injection of B16F10 cells (10^7^ cells/mouse) to the right flank of male athymic nude mice (6 weeks old; Hana Co. Ltd., Busan, Republic of Korea). After tumor cell implantation, the tumor size was measured daily with a vernier caliper. The tumor size was estimated according to the formula of *V* (mm^3^) = (*a*^2^ × *b*)/2, where *V* is the volume, *a* is the width, and *b* is the length of the tumor. When the average tumor size reached 300 mm^3^, the B16F10 *s.c.* tumor-bearing mice were administered with Lipo-IONP/DOX (24 mgFe/kg; 4 mg/kg DOX) via tail vein injection. At 4 h post-administration, the mice were euthanized, and their major organs (heart, lung, liver, spleen, kidney, and tumor) were collected. After digestion, the tissue-distributed Lipo-IONP/DOX was quantified by measuring the Fe contents using ICP-OES. The acquired iron contents were further subtracted by those of the tumors from the control mice, and the percentage of I.D. per g tissue was calculated for each sample.

#### 2.8.3. In Vivo Magnetic Tumor Targeting

The feasibility of magnet-guided tumor targeting of Lipo-IONP/DOX was evaluated in the B16F10 *s.c.* tumor-bearing mice. When the average tumor size reached 300 mm^3^, the mice were divided into 3 groups (N = 5): (1) PBS, (2) Lipo-IONP/DOX, and (3) Lipo-IONP/DOX+MAG. The mice were administered with either PBS or Lipo-IONP/DOX (24 mgFe/kg; 4 mg/kg DOX) via tail vein injection after anesthetization with an *i.p.* injection of ketamine/xylazine mixture. For the Lipo-IONP/DOX+MAG group, an external magnetic field (magnetic field density: 320 mT) was locally applied for 30 min to the tumor region after the drug injection. For the magnetic targeting, a tandem-linked cylindrical neodymium magnet (D48-N52 [6.35 mm diameter × 12.7 mm thickness] and DY0Y0-N52 [51 mm diameter × 51 mm thickness]; K&J Magnetics Inc., Pipersville, PA, USA) was used according to Zhang et al. [12]. After 2 h post-administration, all the mice were euthanized. The tumor tissues were harvested and digested in 1 N nitric acid for 3 days. The digested tumor samples were sent for ICP-OES analyses to quantify the iron contents. The acquired iron contents were further subtracted by those of the tumors from the control mice, and the percentage of injected dose (I.D.) per g tissue was calculated for each sample. In addition, for histological analysis, some of the tumors were collected and fixed in 10% formalin. These tissue samples were dissected (8 µm thick) and embedded in paraffin. The sections were stained with Prussian blue (for IONP detection) and neutral red (for counterstain) to identify the presence of Lipo-IONP/DOX in the tumors [21].

#### 2.8.4. In Vivo Photothermal Activity of Lipo-IONP/DOX

When the average tumor size reached 300 mm^3^, the B16F10 tumor-bearing nude mice were divided into 3 groups: (1) PBS control (N = 5), (2) Lipo-IONP/DOX (N = 10), and (3) Lipo-IONP/DOX+MAG (N = 5). The PBS and Lipo-IONP/DOX (24 mgFe/kg; 4 mg/kg DOX) were administered via tail vein injection, and then the mice were anesthetized with a ketamine/xylazine mixture. For the Lipo-IONP/DOX+MAG group, a magnet was locally applied to the tumor region for 30 min. At 2 h post-administration, the tumors were irradiated with a diode laser (λ = 885 nm) at varying laser powers (0.5–0.95 W). For half of the Lipo-IONP/DOX mice (N = 5), the contralateral skin region was irradiated with the laser instead of the tumor region. The highest temperature of the tumor surface was monitored by using an IR camera (E5, FLIR Systems).

#### 2.8.5. In vivo Evaluation of Efficacy and Toxicity

Nine days after tumor implantation (at day 9), when the average tumor size reached 200 mm^3^, the B16F10 *s.c.* tumor-bearing nude mice were divided into 5 groups (N = 5): (1) PBS-control, (2) DOX (4 mg/kg), (3) Lipo-IONP/DOX (24 mgFe/kg Lipo-IONP; 4 mg/kg DOX), (4) Lipo-IONP/DOX with magnetic targeting (Lipo-IONP/DOX+MAG), (5) Lipo-IONP/DOX with magnetic targeting & PTT (Lipo-IONP/DOX+MAG+PTT). For groups 4 and 5, the mice were anesthetized and, after *i.v*. injection of the Lipo-IONP/DOX, a magnet was locally applied to the tumor regions for 30 min. For group 5, after magnetic field application, at 2 h post-administration, a laser was irradiated to the tumor region for 10 min with 0.7 W power, respectively. These laser powers were chosen to acquire a maximum tumor temperature of about 50 °C. The treatment was carried out three times on Days 9, 11, and 13, and the study continued until the average tumor size of the PBS-control group was above 1500 mm^3^. After the efficacy study was terminated, the mice were euthanized, and the major organs (e.g., tumor, liver, spleen, kidney, and lung) were collected and fixed in 10% formalin. These tissue samples were dissected (8 µm thick) and embedded in paraffin. The sections were stained with hematoxylin and eosin (H&E) to observe nucleic acids and cytoplasms using a previously reported method [22]. In addition, specifically, terminal deoxynucleotidyl transferase dUTP nick end labeling (TUNEL) assay was further carried out for the tumor tissue sections to observe the presence of tumor cell death. The DeadEnd fluorometric TUNEL system (Promega, Madison, WI, USA) was used for labeling apoptotic bodies in tumor sections cover-slipped with a mounting medium, following the manufacturer’s protocol. DAPI dye was used for counterstaining the cell nuclei. The specimens were examined under the DAPI and FITC channel using the Zeiss Axio Observer Z1 fluorescence microscopy (Carl Zeiss MicroImaging GmbH, Jena, Germany).

### 2.9. Statistical Analysis

Data were presented as mean ± standard error of the mean (mean ± SEM). Statistical significant differences among groups were compared by adopting either Student’s *t*-test or 1-way ANOVA (Tukey’s multiple comparison test as post hoc test) (Prism version 8.0, GraphPad, San Diego, CA, USA). Results with *p* < 0.05 were considered statistically significant.

## 3. Results and Discussion

### 3.1. Physicochemical Characterization of Lipo-IONP

**Figure 2:**
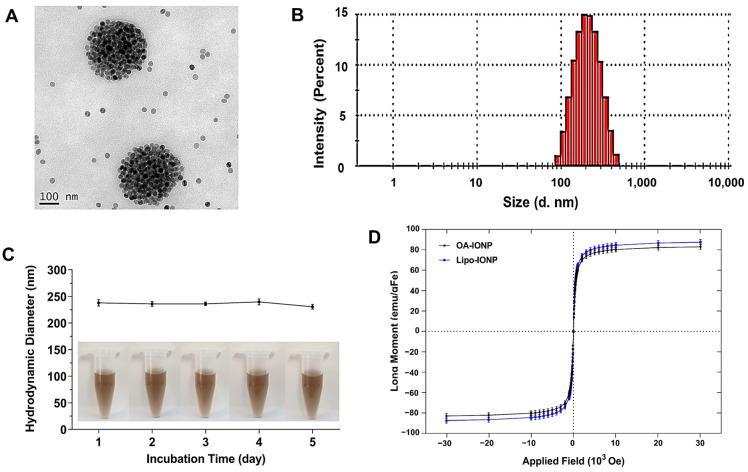
Physical characterization of Lipo-IONP. (**A**) Transmission electron microscopic (TEM) images of Lipo-IONP. (**B**) Hydrodynamic size distribution of Lipo-IONP. (**C**) Size stability of Lipo-IONP. (**D**) Superparamagnetic properties of Lipo-IONP measured by superconducting quantum interference device (SQUID) (Lipo-IONP: liposomal iron oxide nanoparticle).

Lipo-IONP could be successfully synthesized by adopting the thin-film evaporation method. During hydration, non-encapsulated OA-IONP was precipitated as they were insoluble in water and could be readily removed by filtration. The Lipo-IONP prepared by different DPPC-to-DSPE-P2000 ratios (3:1 to 5:1) showed similar morphological and thermodynamic profiles. The Lipo-IONP, identified from the TEM image (Figure 2A), was globular-shaped with sizes of about 30 nm in diameter. They were gathered together to form larger nanoclusters (undissociated by sonication), with mean hydrodynamic sizes of 231.5, 236.3, and 242.1 nm (for L1, L2, and L3), respectively (Figure 2B and Table 1). The surface charge of the Lipo-IONP was negative (−30.5 to −35.1 mV), attributed to the negatively charged DSPE-PEG2000. The transition temperatures of the liposomes prepared by the lipid compositions of L1–L3 were similarly about 47–48 °C, consistent with the lipid mixtures’ theoretical values (Table 1). However, there was a significant difference in the IONP loading efficiency among L1–L3. While the loading efficiency of IONP for L1 was only 34.8%, they were 55.3 and 63.5% for L2 and L3, respectively. (Table 1 and Supplementary Figure S1). These results suggested that PEG may negatively affect the hydrophobic interaction between the OA-IONP and the lipids during the loading process. Similarly, a decrease in drug loading to PEGylated liposomes has often been reported at high incorporation ratios of PEG-conjugated lipids [23,24,25]. Considering the highest loading efficiency, the L3 was chosen for further studies.

**Figure 3:**
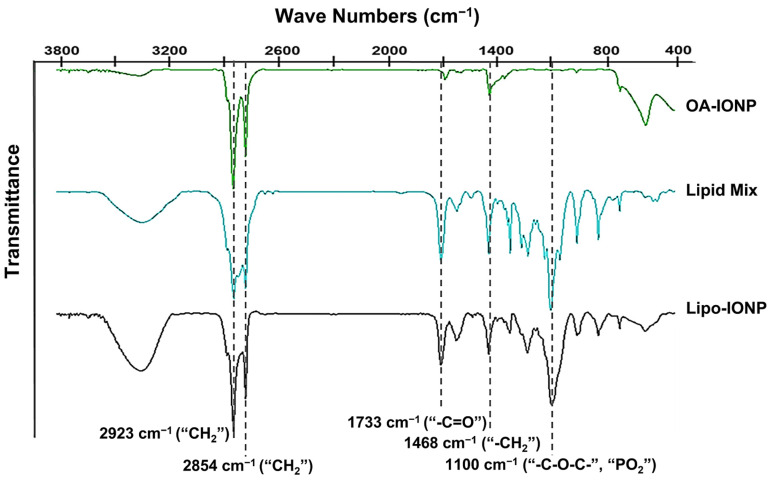
Fourier transform infrared (FT-IR) spectroscopic profiles of Lipo-IONP. The characteristic peaks of the samples are shown in dashed lines. The distinct peaks of the oleic acid composing OA-IONP (at 1733, 2854, and 2923 cm^−1^) and lipid mix (at 1100 cm^−1^) were identified from the FT-IR spectrum of the Lipo-IONP (Lipo-IONP: liposomal iron oxide nanoparticle).

As shown in Figure 2C, the Lipo-IONP stably maintained their sizes for 5 days without the occurrence of apparent aggregation. When the magnetization property of Lipo-IONP was examined by SQUID, as shown in Figure 2D, the magnetization curves clearly showed superparamagnetic characteristics of the Lipo-IONP. The magnetic property of Lipo-IONP (maximum magnetic moment: 87.5 emu/gFe) was nearly equivalent to that of OA-IONP (83 emu/gFe). The measurement of the OA-IONP was also close to the previous report by Clauson et al. [26]. The FT-IR data evidenced the successful lipid coating of the OA-IONP (Figure 3). As seen, the characteristic peaks of OA-IONP and lipid mixtures were both observed from the FT-IR spectra of Lipo-IONP. From the spectrum of OA-IONP, the peaks at 1468, 2854, and 2923 cm^−1^ corresponded to the scissoring, symmetric, and asymmetric stretching vibrations of “CH_2_”, respectively, while the peak at 1733 cm^−1^ was attributed to the “–C=O” stretching vibration of oleic acid [27]. These characteristic peaks of OA-IONP were also observed from both the spectra of lipid mix and Lipo-IONP. However, another characteristic peak at 1100 cm^−1^ attributed to the stretching vibration of “–C–O–C–” and “PO_2_“ of the DPPC and DSPE-P2000 were identified from the spectra of lipid mix and Lipo-IONP [28,29,30]. Overall, these data evidenced the co-presence of DPPC and DSPE-P2000 mix with the OA-IONP in the synthesized Lipo-IONP.

### 3.2. Photothermal Activity of Lipo-IONP

**Figure 4:**
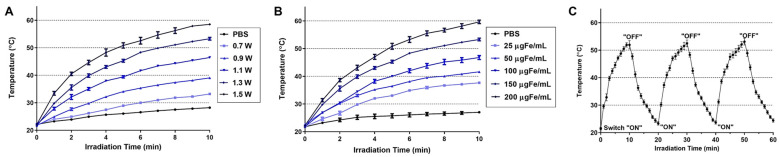
In vitro assessment of the light-to-heat conversion efficiency of Lipo-IONP. Temperature profiles of Lipo-IONP suspensions after laser irradiation (**A**) at different laser powers with fixed particle concentrations (150 μgFe/mL) and (**B**) at different particle concentrations with fixed laser power (1.3 W). (**C**) The stability of the Lipo-IONP’s photothermal activity was evaluated by monitoring the suspension temperature after laser irradiation for three cycles by a switch “on” and “off” mode (10 min for each mode in every cycle) (Lipo-IONP: liposomal iron oxide nanoparticle).

The light-to-heat conversion capacity and photostability of the Lipo-IONP were evaluated in vitro. The data are summarized in Figure 4. When a diode laser was irradiated to the Lipo-IONP suspension, the temperature was risen and reached a plateau at around 10 min (Figure 4A). With a fixed concentration of Lipo-IONP (150 μg Fe/mL), irradiation at a higher laser power raised the suspension temperature to a higher degree. At laser powers of 0.9–1.5 W, the maximum temperatures of the Lipo-IONP suspension reached 40–60 °C. Also, at a fixed laser power (1.3 W), with an increasing concentration of the Lipo-IONP, higher suspension temperatures were achieved (37–60 °C at 25–200 μgFe/mL) (Figure 4B). These results suggested the potential utility of Lipo-IONP in the PTT of cancer. When the laser was applied to the Lipo-IONP suspension by adopting a “switch on-and-off” mode, the maximum suspension temperatures were identical for the three cycles, indicating the photothermal stability of the particles (Figure 4C). This greater photothermal stability may be the highest advantage of the IONP over small molecule-based PTT agents, such as indocyanine green [22].

### 3.3. DOX Loading and Release

**Figure 5:**
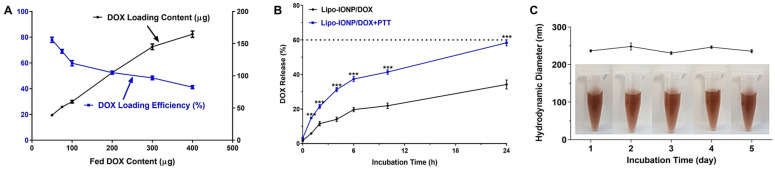
Loading and release of DOX and the size stability of Lipo-IONP/DOX. (**A**) DOX loading content and efficiency of the Lipo-IONP/DOX. (**B**) DOX release profiles with and without laser irradiation. (**C**) Size stability profiles of the Lipo-IONP/DOX. With increasing the DOX fed content, the DOX loading content increased, but, at the same time, the loading efficiency decreased. Based on the results, 200 μg of DOX feeding amount leading to a loading of 105 μg, was chosen for further studies. With laser irradiation above 47 °C (transition temperature of the liposome) for 10 min, a burst release of DOX was observed. A statistically significant difference between the groups was compared by Student’s t-test using Prism software (GraphPad). *** *p* < 0.001 (Lipo-IONP: liposomal iron oxide nanoparticle and Lipo-IONP/DOX: doxorubicin-loaded liposomal iron oxide nanoparticle).

As shown in Figure 5A, higher DOX loading contents were achieved (maximum of 158.7 ± 11.6 μg at 400 μg feeding) by increasing the feed amounts of DOX. However, the DOX loading efficiency was inversely correlated with feed DOX amounts. The DOX loading efficiency decreased from 80.4% to 39.7% by increasing the feed DOX amounts from 50 to 400 μg. Based on the data, we have chosen the feed DOX amounts of 200 μg and targeted 105 μg loading for further studies. According to the drug release study results, the DOX release profiles significantly differed depending upon the laser treatment. As the L3 possessed a favorable transition temperature of 47 °C (theoretically 46.5 °C), a burst release of DOX was observed by heating the sample over 48 °C with laser irradiation. The accumulated DOX release amounts at 24 h post-incubation were 34.2% (without PTT) vs. 58.3% (with PTT) (Figure 5B). At 144 h post-incubation, both groups’ total accumulated drug release amounts were similarly 84%. The average hydrodynamic sizes of Lipo-IONP/DOX were 237, 248, 231, 247, and 236 nm from day 1 to 5 (Figure 5C). Prior to each measurement, the samples were briefly sonicated (3 s at 10% output), which could have slightly affected the size distribution profiles. However, the Lipo-IONP/DOX showed relatively good stability in size for 5 days while kept in the refrigerator.

### 3.4. Cellular Analyses of Lipo-IONP/DOX-Mediated Anti-Cancer Activity

**Figure 6:**
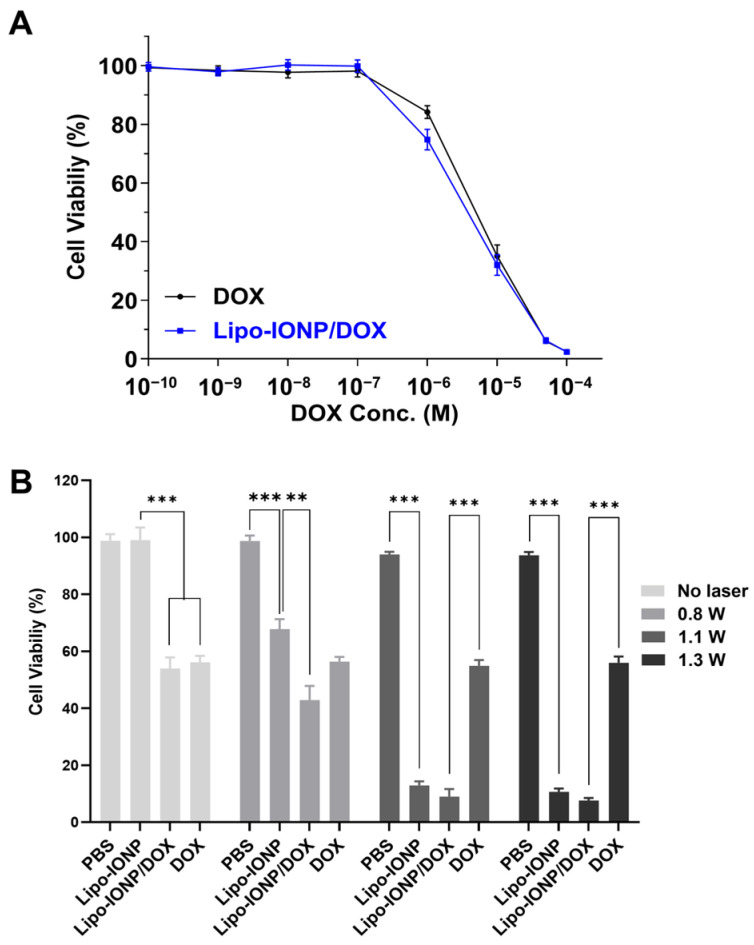
Cytotoxicity of Lipo-IONP/DOX. (**A**) Cytotoxicity profiles of Lipo-IONP/DOX versus DOX alone. (**B**) The viability of cells treated with Lipo-IONP, Lipo-IONP/DOX, and DOX with laser irradiation at different powers. The 0.8, 1.1, and 1.3 W were available to induce maximum medium temperatures of 40, 45, and 50 °C, respectively. The Lipo-IONP/DOX and DOX alone showed similar cytotoxicity levels. However, compared to Lipo-IONP, the Lipo-IONP/DOX elicited greater cytotoxicity. Of note, with laser irradiation at high powers (1.1 and 1.3 W), markedly enhanced cytotoxicity was observed from the cells treated with either Lipo-IONP or Lipo-IONP/DOX. In sharp contrast, the DOX-treated cells showed similar levels of cytotoxicity regardless of the laser application. A statistically significant difference among the groups was compared by 1-way ANOVA with Tukey’s multiple comparison test as the post hoc test using Prism software 9.0.2 (GraphPad). ** *p* < 0.01 and *** *p* < 0.001 (Lipo-IONP: liposomal iron oxide nanoparticle and Lipo-IONP/DOX: doxorubicin-loaded liposomal iron oxide nanoparticle).

The dose-versus-response curves for the cytotoxicity of Lipo-IONP/DOX and free DOX are shown in Figure 6A, and the photothermal effects on the cytotoxicity are shown in Figure 6B. As seen in Figure 6A, the Lipo-IONP/DOX and free DOX exerted concentration-dependent cytotoxicity at similar levels (IC_50_: 4.6(±0.8) vs. 6.1(±1.1) μM). However, the Lipo-IONP alone did not elicit cytotoxicity as high as 1000 μgFe/mL (data not shown). Notably, significantly higher cytotoxic effects were observed from the cells treated with laser after incubation with Lipo-IONP (or Lipo-IONP/DOX) (Figure 6B). The cytotoxicity levels were positively correlated to the laser output. The cell viability levels of Lipo-IONP/DOX were 43, 9.0, and 7.7%, while, for Lipo-IONP, 68, 13, and 11%, at 0.9, 1.1, and 1.3 W, respectively. In sharp contrast, the cells treated with DOX showed similar cytotoxicity levels regardless of the laser application. With laser irradiation at 1.1 or 1.3 W, the Lipo-IONP/DOX provided significantly augmented cytotoxicity than the DOX. Of note, the results revealed that marked anti-cancer effects could be achieved by PTT, specifically above 45 °C. Furthermore, the data also suggested that the combination of PTT and DOX may provide greater therapeutic effects than the PTT or DOX alone.

### 3.5. Pharmacokinetic Profiles

**Figure 7:**
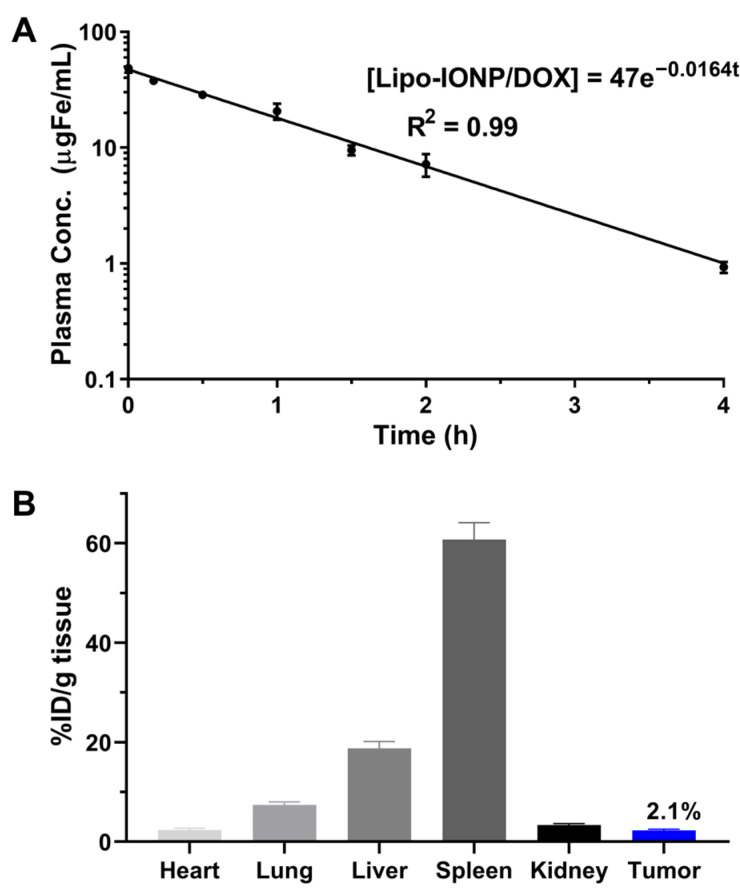
Pharmacokinetics (PK) and biodistribution profiles of Lipo-IONP/DOX in ICR mice. (**A**) Plasma concentration-versus-time profiles of the Lipo-IONP/DOX. (**B**) Tissue distribution of Lipo-IONP/DOX at 4 h post-administration. The PK profiles of the Lipo-IONP/DOX fit a 1-compartment model, and the calculated plasma half-life was 42 min. The tissue distribution data showed that the spleen and liver were the main distribution organs and the tumor content of the Lipo-IONP/DOX determined by ICP-OES was 2.1% ID/g tissue (Lipo-IONP/DOX: doxorubicin-loaded liposomal iron oxide nanoparticle, ICP-OES: inductively coupled plasma optical emission spectroscopy).

After intravenous (*i.v.*) administration of Lipo-IONP/DOX to ICR mice (N = 5), the plasma concentrations of the particles at pre-determined time points were quantified by ICP-OES. The acquired plasma concentration-versus-time profile of Lipo-IONP/DOX is shown in Figure 7A. As seen, the curve for Lipo-IONP/DOX fitted well with the 1-compartment model. The calculated plasma half-life (t_1/2_) of the Lipo-IONP/DOX was 42 min, suggesting that lipid coating and further PEGylation could effectively stabilize the OA-IONP in the bloodstream.

### 3.6. Tissue Distribution, Magnetic Tumor Targeting, and Photothermal Effects in B16F10 S.c. Tumor-Bearing Nude Mice

**Figure 8:**
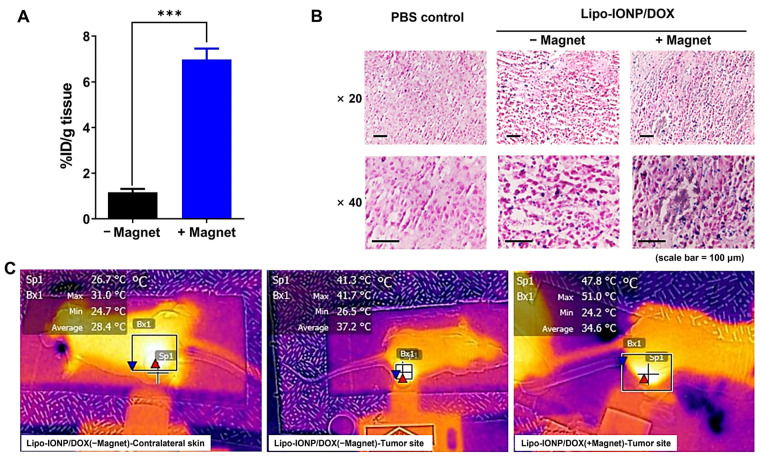
Magnetic tumor targeting of Lipo-IONP/DOX and its photothermal effects in B16F10 *s.c.* allograft tumor mouse model. (**A**) Tumor accumulation profiles of Lipo-IONP/DOX. (**B**) Histological analysis of the tumor distribution of Lipo-IONP/DOX. IONP were stained blue and cells pink by the Prussian blue staining. (**C**) Representative infrared/brightfield overlapped images of Lipo-IONP/DOX administered mice during laser irradiation. When the average tumor size reached 300 mm^3^, the mice were divided into 3 groups (N = 5): (1) PBS, (2) Lipo-IONP/DOX(−Magnet), and (3) Lipo-IONP/DOX(+Magnet). The mice were administered with either PBS or Lipo-IONP/DOX (24 mgFe/kg as Lipo-IONP; 4 mg/kg as DOX) via tail vein injection after anesthetization. For the Lipo-IONP/DOX(+Magnet) group, an external magnetic field was locally applied for 30 min to the tumor region after injection of the Lipo-IONP/DOX. At 2 h post-administration, the tumors were harvested to quantify the Lipo-IONP/DOX contents or histological analysis. To evaluate the in vivo photothermal effects, using the identical animal model, for 2 groups (N = 5): Lipo-IONP/DOX(−Magnet) and (3) Lipo-IONP/DOX(+Magnet), the tumor regions were irradiated with a diode laser (λ = 885 nm) with varying laser powers (0.5–0.95 W) at 2 h post-administration. The highest temperature of the tumor surface (or the contralateral region) was monitored using an IR camera (E5, FLIR Systems). The statistically significant difference in the tumor accumulation amounts between the two groups was compared by Student’s t-test. (GraphPad). *** *p* < 0.001 (Lipo-IONP/DOX: doxorubicin-loaded liposomal iron oxide nanoparticle).

The tissue distribution profiles of Lipo-IONP/DOX in B16F10 *s.c.* tumor-bearing nude mice are shown in Figure 7B. At 4 h post-administration, the major organs were harvested. The Lipo-IONP/DOX was mainly observed in the spleen, liver, lung, kidney, heart, and tumor. Of note, consistent with the previous reports of IONP, the spleen was the highest distribution organ [31]. Specifically, in the tumor, the particles accumulated for an average of 2.1% injected dose (I.D.) per gram of tissue. Figure 8A shows the tumor accumulation profiles of the Lipo-IONP/DOX with and without the application of an external magnet (for 30 min) at the tumor site after administration of the Lipo-IONP/DOX. The mice were then euthanized, and the accumulated Lipo-IONP/DOX was quantified based on the iron contents using ICP-OES. The results showed significantly higher (6.3-fold) particle accumulation in the magnetically-targeted tumors than in the non-targeted ones (6.7 vs. 1.1% I.D./g tissue). Consistent with these measurements, the tumor sections stained with IONP-sensitive Prussian blue dyes showed a markedly higher presence of the particles in the magnetically targeted tumors compared with the non-targeted ones (Prussian blue dye-stained Lipo-IONP/DOX was observed as blue dots in Figure 8B). Figure 8C exhibits mice’s representative IR thermal images during local laser irradiation at the tumor site. Maximum tumor surface temperatures reached with different laser powers (0.5–0.95 W) are summarized in Table 2. As seen, the surface temperatures of the laser irradiation site increased with increasing laser powers. The temperature rise was from 29.1 (at 0 W) to 37.2 °C (at 0.95 W) and from 29.2 to 37.5 °C for the tumors of PBS-control and the contralateral normal skins of the Lipo-IONP/DOX mice, respectively. In sharp comparison, the average tumor temperature of the Lipo-IONP/DOX mice rose from 29.4 to 48.8 °C, and, notably, that of the Lipo-IONP/DOX + MAG mice was increased from 29.1 to 59.8 °C. Based on these results, 0.7 W of laser power (average tumor surface temperature: 50.8 °C) was selected for the efficacy study.

### 3.7. Therapeutic Efficacy and Toxicity

**Figure 9:**
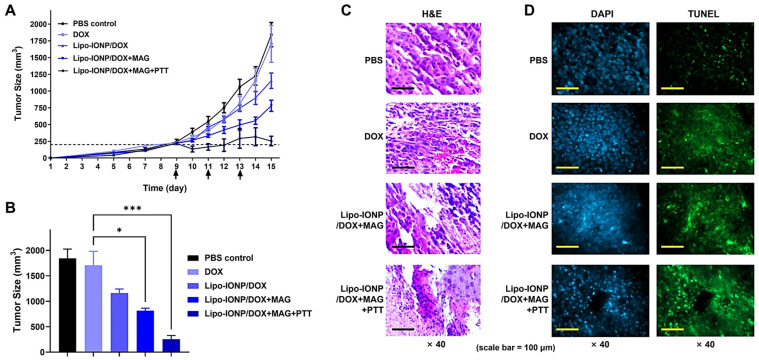
Efficacy study results of the combined chemo-photothermal therapy with Lipo-IONP/DOX in B16F10 *s.c.* allograft tumor mouse model. (**A**) Tumor size profiles along the time. (**B**) Tumor sizes at day 15 when the study was terminated. (**C**) Histological analyses of the tumor tissues. (**D**) TUNEL assay results for the tumor tissues (cells were labeled by TUNEL (green) and nuclei stained by DAPI (blue)). Nine days after tumor implantation (at day 9), when the average tumor size reached 200 mm^3^, the B16F10 *s.c*. tumor-bearing nude mice were divided into 5 groups (N = 5): (1) PBS-control, (2) DOX (4 mg/kg), (3) Lipo-IONP/DOX (24 mgFe/kg as Lipo-IONP; 4 mg/kg as DOX), (4) Lipo-IONP/DOX with magnetic targeting (Lipo-IONP/DOX+MAG), (5) Lipo-IONP/DOX with magnetic targeting & PTT (Lipo-IONP/DOX+MAG+PTT). For groups 4 and 5, the mice were anesthetized and, after *i.v*. injection of the Lipo-IONP/DOX, a magnet was locally applied to the tumor regions for 30 min. For group 5, after magnetic field application, at 2 h post-administration, a laser was irradiated to the tumor region for 10 min with 0.7 W power, respectively. The treatment was carried out three times on days 9, 11, and 13 (shown by the arrows in Figure 9A, and the study continued until the average tumor size of the PBS-control group was above 1500 mm^3^. After the efficacy study was terminated, the mice were euthanized, and the major organs (e.g., tumor, liver, spleen, kidney, and lung) were collected and, after preparation into paraffin-embedded tissue sections, stained with hematoxylin and eosin (H&E) to observe nucleic acids and cytoplasms. The TUNEL assay was further carried out along with DAPI nuclei staining for the tumor tissue sections to observe the presence of tumor cell death. Statistically significant difference among the groups was compared by 1-way ANOVA (Tukey’s multiple comparison test as the post hoc test). * *p* < 0.05 and *** *p* < 0.001 (Lipo-IONP/DOX: doxorubicin-loaded liposomal iron oxide nanoparticle, TUNEL: terminal deoxynucleotidyl transferase dUTP nick end labeling).

The efficacy of Lipo-IONP/DOX-based PTT was evaluated in B16F10 *s.c.* tumor-bearing nude mice. The mice were divided into a total of 5 groups and treated with either (1) PBS, (2) DOX, (3) Lipo-IONP/DOX, (4) Lipo-IONP/DOX with magnetic targeting (Lipo-IONP/DOX+MAG), or (5) Lipo-IONP/DOX with magnetic targeting and PTT (Lipo-IONP/DOX+MAG+PTT), respectively. The results are shown in Figure 9. As seen in Figure 9A, on day 15 (termination day of the study), compared to the PBS-control group (average tumor size: 1840 mm^3^), the DOX treatment at 4 mg/kg provided little therapeutic effects (average tumor size: 1710 mm^3^). In comparison, the Lipo-IONP/DOX could inhibit tumor growth by about 37% (average tumor size: 1170 mm^3^). With magnetic targeting (Lipo-IONP/DOX+MAG), the tumor growth could be inhibited by 56% (average tumor size: 810 mm^3^). However, the Lipo-IONP/DOX+MAG+PTT group showed the highest therapeutic efficacy by marked 84% inhibition of tumor growth.

After the termination of the study, all the mice were sacrificed, and the major organs (e.g., kidney, heart, kidney, liver, and spleen) were harvested and subjected to histopathological analysis. As shown in Figure 9C (H&E staining image), tumor sections from PBS-control and DOX-treated mice showed a well-distributed population of healthy cells. However, some areas of the tumor sections of Lipo-IONP/DOX+MAG showed a loss of cells. Specifically, the tumor section of the Lipo-IONP/DOX+MAG+PTT mice showed not only a marked reduction of the tumor cells but also obvious signs of ongoing cell death processes. As revealed in the histological section, many multinucleated cell formations suggested the occurrence of massive apoptosis. The induction of apoptosis in the tumors of Lipo-IONP/DOX+MAG+PTT-treated mice was further confirmed by the TUNEL assay results (Figure 9D). Tumor sections of Lipo-IONP/DOX+MAG+PTT-treated mice showed significantly more apoptotic bodies (green fluorescent bodies) than those of mice treated with Lipo-IONP/DOX+MAG or DOX only. Few TUNEL-positive cells were found in the tumor sections in the control group mice (PBS only).

**Figure 10:**
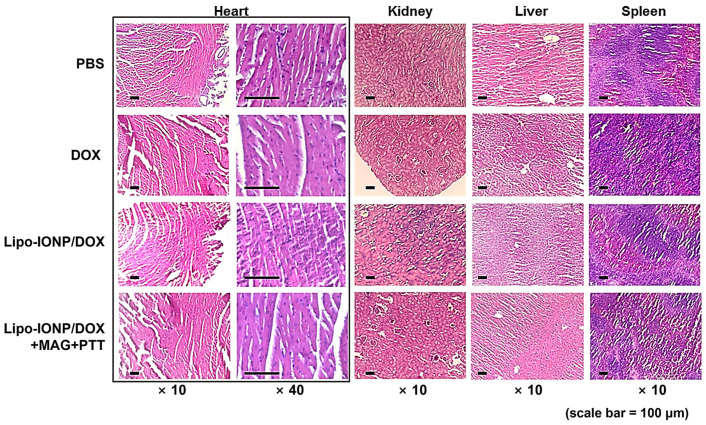
Histological analysis of the major organs after the efficacy study. The images showed no apparent toxicity from any of the major organs of all the experimental groups. Heart, kidney, liver, and spleen tissues were removed from the mice of each group and stained by H&E: mice groups are (1) PBS-control, (2) DOX (4 mg/kg), (3) Lipo-IONP/DOX (24 mgFe/kg as Lipo-IONP; 4 mg/kg as DOX), (4) Lipo-IONP/DOX+MAG+PTT (24 mgFe/kg as Lipo-IONP; 4 mg/kg as DOX). For the Lipo-IONP/DOX+MAG+PTT group, the mice were administered with Lipo-IONP/DOX and then followed by magnetic targeting and photothermal treatment (Lipo-IONP/DOX: doxorubicin-loaded liposomal iron oxide nanoparticle).

Regarding the safety issues, during the efficacy study, the average body weights of the mice were monitored, and the results showed continuous growth with no significant differences among the group (no data shown). Furthermore, histological analyses of the major organs (Figure 10) revealed no apparent toxicity in all the tested groups. Overall, these results demonstrated that the Lipo-IONP/DOX-based PTT might serve as an effective and safe mode of therapy for cancer treatment.

## 4. Conclusions

In this research, we reported the successful synthesis of Lipo-IONP using the thin film evaporation method without help from any stabilizer. The final Lipo-IONP formulation (L3) could be prepared with a high encapsulation efficiency (63.5%). The Lipo-IONP possessed great stability, magneticity, and photothermal activity, as characterized in vitro. Also, with high loading of DOX to Lipo-IONP, laser-induced burst release was available at usually adopted temperatures (>48 °C) for PTT. Furthermore, the Lipo-IONP/DOX could be magnetically targeted to the tumor in vivo, leading to higher photothermal effects. Accordingly, in the efficacy study with the B16F10 *s.c.* allograft tumor mice model, the combined chemo-photothermal treatment with Lipo-IONP/DOX elicited the most significant effects in tumor growth inhibition. Overall, this research demonstrated that the Lipo-IONP/DOX could serve as an effective and safe agent for combined chemo-photothermal cancer therapy.

## Figures and Tables

**Table 1:** Physical characterization of Lipo-IONP synthesized with different lipid ratios.

Lipo-IONP	DPPC:DSPE-P2000	Hydrodynamic Size (nm)	PDI	Zeta Potential (mV)	Iron Loading Content (μgFe)	Transition Temperature (°C)
L1	3:1	231.5 (±6.6)	0.25	−30.5 (±0.4).	348 (±43)	48.79
L2	4:1	236.3 (±1.5)	0.24	−34.8 (±0.6)	553 (±74)	47.98
L3	5:1	242.1 (±1.2)	0.26	−35.1 (±0.2)	635 (±64)	47.04

**Table 2:** Maximum tumor temperature profiles of Lipo-IONP/DOX-treated mice with photothermal treatment.

Laser Power (W)	Surface Temperature of the Laser Irradiation Site (°C)			
	PBS-Control	Lipo-IONP/DOX (−Magnet)		Lipo-IONP/DOX (+Magnet)
	Tumor	Contra-Lateral Normal Skin	Tumor	Tumor
0.95	37.2	37.5	48.8	59.8
0.9	36.1	36.6	48.1	59.2
0.8	34.8	35.2	46.2	54.9
0.7	32.5	33.1	42.7	50.8
0.6	31.6	32.2	41.6	45.6
0.5	29.3	29.5	39.5	41.5
0	29.1	29.2	29.4	29.1

## Data Availability

Not applicable.

## Supplementary Materials

The following supporting information can be downloaded at: https://www.mdpi.com/article/10.3390/pharmaceutics15010292/s1, Figure S1: The differential scanning calorimetry (DSC) measurement results of Lipo-IONP synthesized with different lipid ratios; (**A**) L1, (**B**) L2, (**C**) L3. These Lipo-IONP formulations were prepared with the ratios of DPPC:DSPE-P2000 of 3:1, 4:1, or 5:1, respectively.
