# Supplementary material for: Liposomal Iron Oxide Nanoparticles Loaded with Doxorubicin for Combined Chemo-Photothermal Cancer Therapy
Source: Pharmaceutics. 2023 Jan 15;15(1):292. doi: 10.3390/pharmaceutics15010292 (PMC9860715; doi:10.3390/pharmaceutics15010292)
Supplement: Supplementary file 1 [file pharmaceutics-15-00292-s001.zip › pharmaceutics-2091771-supplementary.pdf]

Supplemental Material: Liposomal iron oxide nanoparticles loaded with doxorubicin for combined chemo-photothermal cancer therapy

Taehoon Park, Reeju Amayta, Kyoung Ah Min * and Meong Cheol Shin *

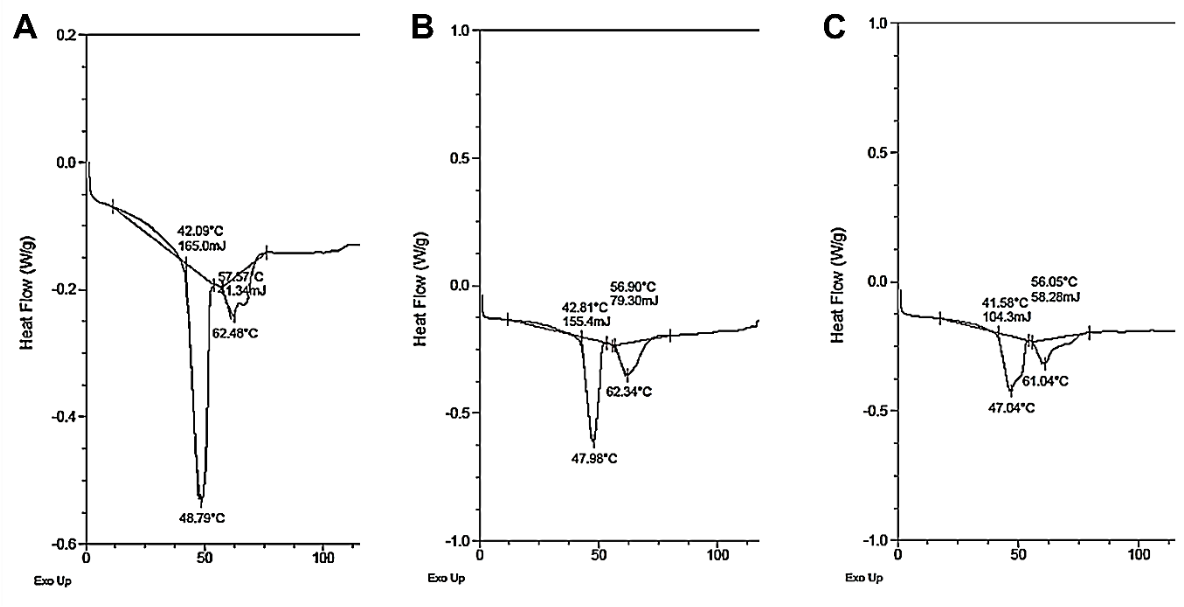

Figure S1. The differential scanning calorimetry (DSC) measurement results of Lipo-IONP synthesized with different lipid ratios; (**A**) L1, (**B**) L2, (**C**) L3. These Lipo-IONP formulations were prepared with the ratios of DPPC:DSPE-P2000 of 3:1, 4:1, or 5:1, respectively.
